# Disability risks in past populations: Sweden from the 1800s until 1959

**DOI:** 10.1093/eurpub/ckac130.050

**Published:** 2022-10-25

**Authors:** L Vikström, J Junkka, E Häggström Lundevaller

**Affiliations:** 1 Department of Historical, Philosophical and Religious Studies, Umeå University, Umeå, Sweden; 2 Centre for Demographic and Ageing Research, Umeå University, Umeå, Sweden

## Abstract

**Background:**

While diseases in contemporary and past populations are thoroughly studied, the knowledge about disability and the risks of getting it is poor. Like diseases, disabilities increase with growing age affecting primarily elderly groups. Whether this notion holds historically and for other groups at risk for disability and differences over time is not known. This study estimates the disability risks in Swedish populations c. 1800-1959 by age, sex and disability type (sensory, physical, mental).

**Methods:**

We use data on two historical populations in the 1800s (N = 36,500; 550 with disability) and 1900-1959 (N = 194,500; 4,700 with disability) drawn from digitized parish registers reporting socio-economic and demographic characteristics over lifetime and on disabilities. Cox proportional regressions estimate disability risks across time by group (age, sex, disability type).

**Results:**

Our preliminary results based on unadjusted estimates from 1900-1959 suggest that the disability risks doubled or more. In the 1950s, women had 2.6 times higher risk than 50 years before, while it was 2.0 for men. The major rise started in the 1930s (Men 1.51; Women: 1.67), and grew in the 1940s (Men 1.80; Women: 2.14). Next, we will assess these risks by group and in the 1800s.

**Conclusions:**

From 1900-1959, Swedish populations experienced consistently higher disability risks, which doubled for men and almost tripled for women. These risks increased while improvements in public health and economic growth would subsequently make Sweden internationally known as a modern welfare state. That health improvements did not reduce the disability risks but the reverse, was possibly due to higher recognition or labeling of disabilities.

**Key messages:**

- Our study is unique in providing long-term results on populations at risk for disability while public health improved.
- Public concerns to confine disabled people for treatment in the early welfare era increased the disability risks beside longer life expectancy.

